# The tortoise and the hare revisited

**DOI:** 10.7554/eLife.01233

**Published:** 2013-09-03

**Authors:** Natalia L Kononenko, Arndt Pechstein, Volker Haucke

**Affiliations:** 1 **Natalia L Kononenko** is in the Department of Molecular Pharmacology and Cell Biology, Leibniz Institut für Molekulare Pharmakologie (FMP) and Neurocure Cluster of Excellence, Charité Universitätsmedizin Berlin, Berlin, GermanyKononenko@fmp-berlin.de; 2 **Arndt Pechstein** is in the Department of Molecular Pharmacology and Cell Biology, Leibniz Institut für Molekulare Pharmakologie (FMP) and Neurocure Cluster of Excellence, Charité Universitätsmedizin Berlin, Berlin, GermanyPechstein@fmp-berlin.de; 3 **Volker Haucke** is in the Department of Molecular Pharmacology and Cell Biology, Leibniz Institut für Molekulare Pharmakologie (FMP) and Neurocure Cluster of Excellence, Charité Universitätsmedizin Berlin, Berlin, GermanyHaucke@fmp-berlin.de

**Keywords:** synaptic vesicle endocytosis, optogenetics, time-resolved electron microscopy, high-pressure freezing, synaptic vesicle exocytosis, active zone, *C. elegans*

## Abstract

Optogenetics and electron microscopy reveal an ultrafast mode of synaptic vesicle recycling, adding a new twist to a 40-year-old controversy.

**Related research article** Watanabe S, Liu Q, Davis MW, Hollopeter G, Thomas N, Jorgensen NB, Jorgensen EM. 2013. Ultrafast endocytosis at *Caenorhabditis elegans* neuromuscular junctions. *eLife* **2**:e00723. doi: 10.7554/eLife.00723

**Image** An electron micrograph showing invagination of the plasma membrane (arrow) just 20 ms after stimulation

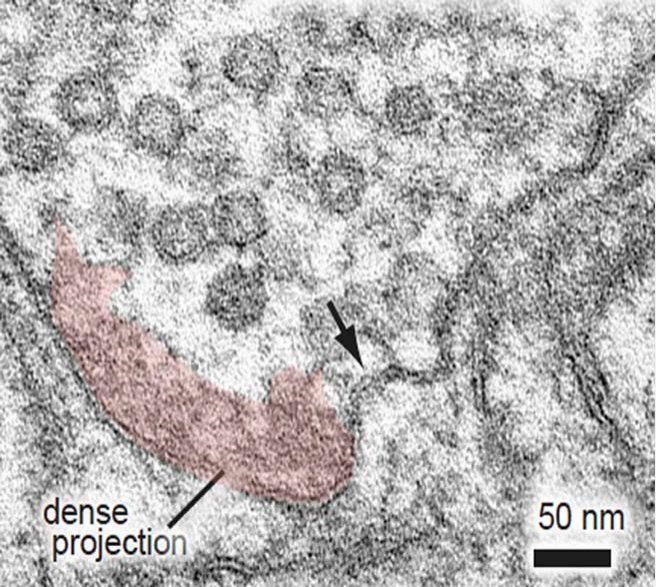

From sensory perception to learning and memory, the functioning of the nervous system is dependent upon communication between neurons, which is mediated by chemical neurotransmission at synapses. Vesicles loaded with neurotransmitter fuse with the presynaptic plasma membrane and release their contents in a process known as exocytosis. To avoid depletion of the supply of vesicles, those that have been released are then recycled through a process called endocytosis. But despite the identification of a plethora of proteins involved in endocytosis, the precise pathway by which synaptic vesicles are recycled has remained a matter of debate.

The most fundamental challenge facing those who attempt to identify this pathway is the transient nature of the structures that form during endocytosis. In the early 1970s, Heuser and Reese used rapid tissue freezing paired with conventional electron microscopy to try to examine these structures. By subjecting frog neuromuscular synapses to prolonged high-frequency stimulation, they obtained evidence that vesicle recycling occurs via clathrin-mediated endocytosis (Heuser and Reese, 1973). Their results formed the basis of a model according to which synaptic vesicles collapse fully into the plasma membrane and are subsequently recycled through the formation of membrane ‘pits’, which become surrounded by a coat protein called clathrin and bud off as new vesicles. This is a slow process, taking 10 to 20 s, and occurs at sites distant from the active zone where vesicles release their transmitter (Figure 1A).

**Figure 1.**
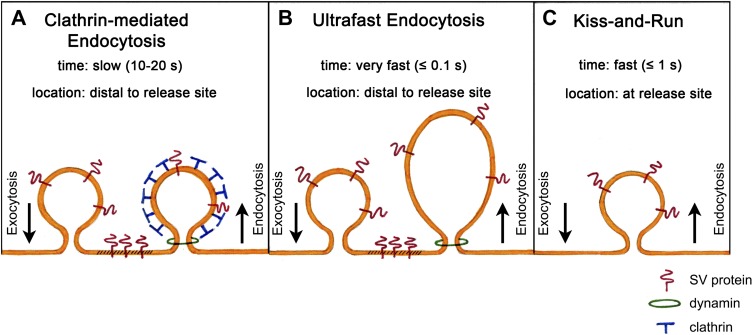
Models of synaptic vesicle endocytosis. (**A**) In clathrin-based endocytosis, synaptic vesicles collapse fully into the plasma membrane, before being retrieved via a slow process (taking about 10–20 s) mediated by the coat protein clathrin. This occurs at sites distant from the site of fusion. (**B**) Watanabe et al. propose a new ultrafast mechanism for synaptic vesicle recycling. This process takes roughly one tenth of a second (or less) and involves the formation of large endocytic ‘pits’ devoid of a clathrin coat. Note that both (**A**) and (**B**) require the GTPase dynamin to pinch off newly formed endocytic vesicles from the plasma membrane. (**C**) In the ‘kiss-and-run’ model, synaptic vesicles form a transient pore through which they release their neurotransmitter contents, and are then rapidly recycled (in about a second) at the site of fusion without being absorbed into the membrane.

Surprisingly, however, another group who were using the same sample preparation, but much lower frequencies of electrical stimulation, failed to observe clathrin-coated intermediates (Ceccarelli et al., 1973). Their results suggested that vesicles do not collapse fully into the membrane, but instead form a transient pore through which they release their neurotransmitter. The pore then reverses, enabling the vesicles to be rapidly recycled at their current location. This process, which has been termed ‘kiss-and-run’ (Figure 1C), takes less than a second and is thus much faster than clathrin-mediated endocytosis. More than 40 years later, the debate about which of these models is correct is still ongoing.

Now, in *eLife*, Erik Jorgensen and co-workers—including Shigeki Watanabe as first author—report a third mechanism for vesicle recycling, which involves neither ‘kiss-and-run’ nor clathrin, and which operates outside the active zone on an ultrafast time scale. To uncover this pathway, Watanabe et al.—who are based at the University of Utah—combined the use of *C. elegans* that had been genetically modified to express a light-sensitive protein called channelrhodopsin, with rapid high-pressure freezing electron microscopy. This approach made it possible to control stimulation and freezing on a millisecond time scale, enabling the team to obtain snapshots of exocytosis and endocytosis.

They found that a single light stimulus triggers vesicles docked with the active zone membrane to undergo exocytosis within 20 ms. These fusion events are followed within 50–100 ms by the appearance of membrane invaginations much larger than a synaptic vesicle, and lateral to the centre of the fusion site. Similar structures are also seen after about 1 s at more distal sites close to the junctions with other cells. By contrast, clathrin-coated intermediates are rarely observed. Given that the appearance of these large endocytic structures is dependent upon stimulation and the prior occurrence of exocytosis, Watanabe et al. conclude that vesicle endocytosis in intact *C. elegans* neuromuscular synapses occurs via a novel ultrafast mechanism (Figure 1B).

The formation of these large endocytic structures in *C. elegans* requires the GTPase enzyme dynamin, consistent with other reports (Dittman and Ryan, 2009). However, the remaining components involved in forming these structures are unknown. Possible candidates include BAR domain proteins such as endophilin (Llobet et al., 2011; Milosevic et al., 2011), actin, and actin regulatory factors, but further studies will be needed to confirm this.

Another key question is how this new mechanism can be reconciled with previous experiments that suggested a key role for clathrin-mediated endocytosis in vesicle recycling (Dittman and Ryan, 2009). One possibility is that synapses may have evolved separate mechanisms for retrieving the membranes of recently released vesicles, and for reforming vesicles from endocytosed material. This is consistent with the observation that vesicle recycling at highly active synapses involves the formation of endosomal intermediate structures—raising the possibility that synaptic vesicles could then be regenerated from these intermediates through clathrin-dependent budding steps. Watanabe et al. may have selectively activated this rapid clathrin-independent membrane retrieval process. In this scenario, clathrin would instead be required at some later stage to turn endosomes or other endocytic structures into synaptic vesicles.

A second possibility is that mechanisms of endocytosis may vary between species more than previously thought. In the most extreme scenario, ultrafast endocytosis could be a special feature of *C. elegans* neuromuscular synapses. This would be consistent with the fact that *C. elegans* with mutations in the clathrin heavy-chain gene show a surprisingly normal presynaptic ultrastructure (Sato et al., 2009). However, mutations in other *C. elegans* genes linked to clathrin-mediated endocytosis such as AP-2 induce severe presynaptic defects (Gu et al., 2013), suggesting that at least some form of clathrin-mediated endocytosis is operational in worms.

A third possibility is that mechanisms of endocytosis may have been adapted to cope with the wide range of frequencies over which neurons can operate. While few if any neurons will encounter single stimuli, many cell types can fire at frequencies of up to 20 Hz for many seconds (Zhang et al., 2013). Furthermore, the hippocampus has been shown to contain a population of pyramidal neurons that can fire bursts of action potentials at more than 50 Hz (Harvey et al., 2009), while some central synapses such as the calyx of Held can reach firing rates of more than 500 Hz (Kopp-Scheinpflug et al., 2008). It thus seems likely that neurons can capitalize on more than one mechanism of endocytosis depending on their firing rate.

Irrespective of these considerations, and although the precise role of clathrin at vertebrate synapses remains to be determined, the work of Watanabe et al. represents an important methodological breakthrough and a key step forward with respect to our understanding of synaptic vesicle endocytosis.
